# Prevalence of presumed ocular tuberculosis among pulmonary tuberculosis patients in a tertiary hospital in the Philippines

**DOI:** 10.1186/1869-5760-3-1

**Published:** 2013-01-03

**Authors:** Leon Paolo R Lara, Vicente Ocampo

**Affiliations:** 1 Department of Ophthalmology, Veterans Memorial Medical Center, North Avenue, Diliman, Quezon City, 1101, Philippines

**Keywords:** Presumed ocular tuberculosis, Prevalence, Anti-tubercular therapy, Extra-pulmonary TB, Anterior uveitis, Posterior uveitis

## Abstract

**Background:**

The objective of this study was to determine the prevalence of presumed ocular tuberculosis among diagnosed pulmonary tuberculosis patients in a tertiary government hospital in the Philippines and determine its common presentation in the population. This was a cross-sectional study in which 103 patients who were labeled to have active pulmonary tuberculosis underwent history and ocular examination prior to anti-tubercular therapy. The diagnosis of presumed ocular tuberculosis was made when clinical signs of tuberculosis (TB) uveitis were found in the participants. Lesions were documented and tallied, after which statistical analysis was performed.

**Results:**

Seven out of the 103 pulmonary TB patients (6.8% prevalence: 95% CI 2.78% to 13.5%) included in the study showed signs of ocular inflammation. There was no sex and age predilection between those with presumed ocular TB and those without. Posterior uveitis alone was observed in three of the patients (two cases of retinal vasculitis and one case of choroidal tubercle). Non-granulomatous anterior uveitis with posterior synechiae alone was observed in two patients. One patient had combined non-granulomatous anterior uveitis with posterior synechiae and choroidal tubercle. One had combined granulomatous anterior uveitis with posterior synechiae and choroidal tubercle. Intermediate uveitis was not noted among the patients.

**Conclusions:**

Presumed ocular tuberculosis should be considered among patients with diagnosed pulmonary tuberculosis. Common ocular lesions found in the study include choroidal tubercle and non-granulomatous anterior uveitis with posterior synechiae.

## Background

According to the World Health Organization, the Philippines ranks fourth in the world for the number of cases of tuberculosis (TB) and has the highest number of cases per head in Southeast Asia. Almost two thirds of Filipinos have TB, and up to five million people are infected yearly [1], making it a major public health concern in the country. TB in the Philippines ranked fifth in the 10 leading causes of death and fifth in the 10 leading causes of illness, with an incidence reported to be 6.3 per thousand per year (culture positive) and 2.6 per thousand per year (smear positive) [2]. The increased incidence has economic repercussions not only for the patient's family, but also for the country, with most TB patients belonging to the economically productive age group (15 to 54 years old) [1].

Though more commonly infecting the pulmonary system, it can also manifest as extra-pulmonary TB (EPTB) affecting the gastrointestinal, skeletal, cardiac, genitourinary, and nervous systems including the eye. Diagnosis of these extra-pulmonary forms is difficult and is often determined by the exclusion of other conditions [3]. Some report that it now constitutes a greater proportion of all patients with TB, especially in immunocompromised individuals and the elderly.

TB in the eye can manifest in a myriad of ways, and the definitive diagnosis can be daunting due to the difficulty of getting ocular samples for microbiologic or histologic evaluation. High awareness of ocular manifestations is a must for an ophthalmologist as he or she may be the first to diagnose TB [4]. A review by Gupta et al. [5] last 2007 updated the clinical spectrum, laboratory investigation, and diagnostic criteria that would assist in the diagnosis of presumed or confirmed intraocular TB so that anti-tuberculous therapy (ATT) can be initiated on a rational basis.

Ocular TB has always been considered rare, yet its prevalence has varied widely across time, patient populations, and geography. Some studies include rates of ocular involvement among patients with pulmonary TB (PTB). Donahue in 1967 reported a prevalence of ocular TB of 1.46% in 10,524 patients from a tuberculosis sanitarium in the USA [6]. A prospective study of Bouza et al. from Spain reported in 1997 examined 100 randomly chosen patients with proven systemic tuberculosis and found ocular involvement in 18 patients (18%) [7]. In Malawi, Africa, a 2.8% prevalence of choroidal granuloma in 109 patients with fever and tuberculosis was reported in a prospective study in 2002 [8]. Biswas and Badrinath examined 2,010 eyes of pulmonary TB patients and found a 1.39% ocular involvement [9].

Other studies include data about ocular TB as a fraction of uveitis cases. It has been estimated to be under 1% in the USA, 4% in China, 6% in Italy, 7% in Japan, and 16% in Saudi Arabia [10]. A Southeast Asian neighbor, Thailand, reported a 2.2% systemic TB involvement [11].

## Results

There were 103 patients who were recruited for the study and who underwent an ocular examination. The mean age was 51.5 years (range 5 to 88), and 62% were male. None of those found to have presumed ocular TB (POTB) presented with ocular findings on both eyes.

### Posterior findings

Majority of ocular findings of those found to have POTB (five eyes of seven people) were located in the posterior segment. Three eyes had a choroidal nodule. There were two cases of vascular sheathing consistent with retinal vasculitis, one having a large number of discrete, mostly peripapillary, blot hemorrhages. During the 4-week follow-up period of the three patients with choroidal tubercles, all showed partial clinical resolution with institution of ATT. The two patients exhibiting retinal vasculitis were lost to follow-up.

### Anterior, intermediate, and other systemic findings

Of the seven patients with presumed ocular TB, four had anterior segment involvement. Three exhibited non-granulomatous anterior uveitis with posterior synechiae, one of whom had an incidental chronic peripheral corneal degeneration on the involved eye. Cervical lymphadenopathy was found in two of these four patients.

One presented as granulomatous anterior uveitis with posterior synechiae and severe vitritis which was eventually managed with pars plana vitrectomy. Polymerase chain reaction (PCR) testing of vitreous aspirate yielded a negative result.

All anterior uveitic lesions showed at least partial clinical resolution with institution of ATT. No signs of intermediate uveitis were found. Results are summarized in Table 1.

**Table 1:** Profile of patients labeled to have presumed ocular TB

**Patient**	**Age (years)**	**Sex**	**Findings**	**VAi**	**VAf**	**ATT response after 4 weeks**
1^a^	66	M	OS: non-GAU with posterior synechiae, choroidal tubercle; grade 2 cataract, L-cervical lymphadenopathy	0.1/0.050	0.1/0.1	Partial
2^a^	68	M	OS: GAU, choroidal tubercle, posterior synechiae	0.4/0.025	0.4/0.025	Partial
3	57	F	OS: choroidal tubercle	0.67/0.67	0.67/0.67	Partial
4^a^	36	F	OS: non-GAU, posterior synechiae, L-cervical lymphadenopathy	0.67/0.40	0.67/0.50	Partial
5	77	M	OS: retinal vasculitis	0.40/0.40	Lost to follow-up	
6	70	F	OD: non-GAU, posterior synechiae	1/1	1/1	Partial
7	67	M	OS: retinal vasculitis	0.40/0.67	Lost to follow-up	

Multiple response: Patients 1 and 2 had combined findings in the anterior and posterior segments of the eye; VAi, best-corrected visual acuity (BCVA) prior to ATT; VAf, BCVA after 4 weeks of ATT; GAU, granulomatous anterior uveitis; OS, left eye; OD, right eye; ^a^IOP of involved eye significantly lower (> 5 mmHg difference).

Seven out of the 103 pulmonary TB patients (6.8% prevalence: 95% CI 2.78% to 13.5%) included in the study showed signs of ocular inflammation. There was no statistically significant difference between the age of those with POTB and those without (*p* = 0.181; Table 2). Though there were more men (five cases) compared to women (two cases) who had ocular lesions, this was not statistically significant (*p* = 0.707).

**Table 2:** Age distribution of study participants

**Age (years)**	**No ocular TB**		**With ocular TB**		**Total**	
	**No.**	**%**	**No.**	**%**	**No.**	**%**
≤ 10	2	2.08	0	-	2	1.94
11 to 20	2	2.08	0	-	2	1.94
21 to 30	11	11.46	1	14.29	12	*11.65*
31 to 40	12	12.50	0	-	12	*11.65*
41 to 50	11	11.46	2	28.57	13	*12.62*
51 to 60	32	33.33	3	42.86	35	*33.98*
61 to 70	17	17.71	1	14.29	18	17.48
71 to 80	5	5.21	0	-	5	4.85
81 to 90	4	4.17	0	-	4	3.88
Total	96	100.00	7	100.00	103	100.00
Mean ± SD	50.93±17.406		60.00±13.760		51 to 544±17.28	
Range	5 to 88		36 to 78		5 to 88	

*p* = 0.181; independent *t* test.

## Discussion

We labeled a patient to have ocular TB in this study based on the proposed diagnostic criteria for presumed ocular TB by Gupta et al. [5] (i.e., a known clinical sign of ocular TB with a positive systemic finding such as a tuberculous lesion on CXR). Though the PCR result done in one of the seven labeled to have POTB was negative, it still does not rule out possible ocular TB, only having a reported maximal sensitivity of 66.6% [12].

Ocular TB is one manifestation of EPTB. It can adversely affect the quality of life of people by threatening vision. Of the 103 pulmonary TB patients in the study, seven (6.8%) showed signs of ocular inflammation. There are probably more cases we were unable to detect because we did not examine EPTB patients. This rate is higher than the 1.39% to 1.46% ocular involvement found in other studies [6,9]. It can expand the knowledge base regarding the epidemiology of POTB and can contribute to greater awareness on the condition.

Ocular TB is not easy to diagnose because most of the time there is no concurrent active systemic TB. Notable in our findings was the unilateral presentation of all patients labeled as POTB, concurring with the reports of some authors that ocular TB is usually unilateral [13]. However, the absence of a single manifestation of POTB further compounds the difficulty in recognizing the disease. In our study, as with other reports [14], posterior segment lesions were the predominant finding in patients with POTB. This would be a logical finding in our diagnosed PTB patient population since choroidal tubercles and retinal vasculitis indicate hematogenous seeding of bacilli. EPTB mainly results from reactivation of a tuberculous focus after hematogenous dissemination or lymphogenous spread from a primary, usually pulmonary, focus [15].

The amount of TB burden necessary in the lungs to produce EPTB has not yet been quantified. TB affects other sites of the body other than the lungs and eyes. One recently published study found that among the total of 2,161 TB infection cases, 705 (32.6%) were EPTB, 1,186 (54.9%) were PTB, 106 (4.9%) were disseminated TB, and 164 (7.6%) were concurrent EPTB-PTB. Most common sites of EPTB they found were in pleural (41.1%) and lymphatic (30.6%) tissues followed by genitourinary (7%), bone/joint (5.8%), cutaneous (4.5%), meningeal (4.1%), peritoneal (2.6%), and gastrointestinal (2%) [16]. In our study, we incidentally detected two cases of cervical lymphadenopathy out of the seven detected POTB cases.

One study conducted a multivariate analysis determining risk factors for developing EPTB relative to PTB, and they found that female gender and older age are associated with EPTB [16]. Our male POTB patients outnumbered the females (4:3). However, we found a relatively higher mean age in our POTB cluster, with six of the seven patients belonging to the 41- to 70-year-old age group. We found the mean age of those with POTB higher (60.00 ± 13.760 years old) than those without ocular findings (50.93 ± 17.406 years old). One East Asian study found increasing longevity of their population and the high rate of TB in their elderly as important factors contributing to their persistent high rate of TB [17]. Old age has indeed been cited to be a risk factor for EPTB since the immune system can be weaker in the elderly [16,18].

The study is limited by a lack of investigations such as fluorescein angiography, indocyanine green angiography, or ocular coherence tomography. Confounders that compromise the immune system were not controlled in the analysis (e.g., DM, HIV). Effect modifiers such as cataract were not controlled. Future studies could look at the clinical/radiological spectrum of PTB cases associated with POTB. Since active PTB can easily be genotyped, genotypic profiling of these cases can also be done.

## Conclusions

Putting things together, an ocular examination before ATT in newly diagnosed TB patients may be beneficial in our setting for the following reasons: the relatively high (6.8%) prevalence of ocular involvement in TB patients found, the possibility of blindness caused by POTB lesions, and the potential toxicity of some ATT drugs. Ultimately, the ophthalmologist and internist should increase their awareness and understanding of TB and its possible ocular involvement because the disease is curable and blindness is preventable [7].

## Methods

The study recruited patients diagnosed to have active PTB from August 2010 to September 2011 at a tertiary government hospital in the Philippines. Patients with respiratory (cough > 2 weeks, hemoptysis, chest pain, breathlessness, etc.) or constitutional symptoms (fever, night sweats, fatigue, loss of appetite, etc.) were seen and examined by the hospital's pulmonology service. A chest X-ray is requested, and if a tuberculous lesion was found, three sputum samples examined for acid-fast bacilli (AFB) were requested. Patient was labeled to have active pulmonary TB when AFB smear was positive. If doubt existed about TB presence due to negative AFB smear result, the hospital's TB Diagnostic Committee evaluated the case to judge whether to label it as active or not.

All patients diagnosed to have active pulmonary TB were referred to the hospital's Department of Ophthalmology prior to start of ATT, and a single examiner evaluated the patients. They were examined for best-corrected visual acuity, intraocular pressure, eye movements, anterior segment pathology, and pupillary reactions. The fundus was examined by indirect ophthalmoscopy. Patients with history of ocular trauma and previously diagnosed retinal or optic nerve diseases were excluded from the study.

Signs of TB uveitis were searched for, namely, non-granulomatous anterior uveitis with posterior synechiae, granulomatous anterior uveitis, iris nodules, ciliary body tuberculoma, granulomatous intermediate uveitis, intermediate organizing exudates, choroidal tuberculoma, subretinal abscess, serpiginous-like choroiditis, retinitis/vasculitis, optic neuropathy, and endophthalmitis. To label a subject as having POTB, we employed the Diagnostic Criteria of Ocular TB proposed by Gupta et al. [5] (Additional file 1). All positive findings were re-assessed and confirmed by a single uveitis specialist. Patients whose first examination was not suggestive of ocular involvement were not evaluated further. Patients with evidence of ocular TB involvement were followed-up after 4 weeks and were labeled to have partial response if showing clinical improvement of ocular lesions during this time period.

A target number of 101 subjects was set on a 95% confidence level and power was set at 80%, and relative error of 15% and assumed ocular TB prevalence of 18% among diagnosed tuberculosis patients were based on the report by Bouza et al. [7]. Frequency of findings was tallied, and significance was assessed by various statistical analyses with alpha set at 0.05. A hospital research committee approved the study, and the tenets of the Declaration of Helsinki were observed.

## Competing interests

The authors report no conflicts of interest. The authors alone are responsible for the content and writing of the paper.

## Authors’ contributions

PL carried out the coordination with the pulmonology department as well as the eye exam of the participants. He also drafted the manuscript. VO examined participants suspected to have presumed ocular TB and was the one to confirm a patient as having presumed ocular TB. He also gave invaluable expert advice throughout the course of the study. Both authors read and approved the final manuscript.

## Supplementary Material

Additional file 1 **Proposed diagnostic criteria of ocular TB by Gupta et al. **[5]**.** Click here for file
