# Supplementary material for: Prevalence of presumed ocular tuberculosis among pulmonary tuberculosis patients in a tertiary hospital in the Philippines
Source: J Ophthalmic Inflamm Infect. 2013 Jan 3;3:1. doi: 10.1186/1869-5760-3-1 (PMC3589205; doi:10.1186/1869-5760-3-1)
Supplement: Additional file 1 — Proposed diagnostic criteria of ocular TB by Gupta et al. [5]. [file 1869-5760-3-1-S1.doc]

**Proposed Diagnostic Criteria of Ocular TB by Gupta et al. [5]**

_________________________________________­­­­­­­­­­­­­­­­­­­­­­­____

CONFIRMED (DEFINITIVE) OCULAR TB

Any one or more of the clinical signs (1) of TB uveitis in combination with any of the positive tests for ocular TB (2)

PRESUMED OCULAR TB

Any one or more of the clinical signs (1) of TB uveitis in combination with any of the positive tests for Systemic investigation of TB (3); *or*

Any one or more of the clinical signs (1) of TB uveitis in combination with a positive therapeutic trial (5) with exclusion of other uveitis entities (4).

1. *Clinical Signs*

2. *Ocular Investigations*

a. Demonstration of AFB by microscope or culture of M. tuberculosis from the ocular fluids.

b. Positive polymerase chain reaction from ocular fluids for IS 6110 or other conserved sequences in M. tuberculosis genome.

3. *Systemic Investigations*

a. Positive Mantoux reaction.

b. Evidence of healed or active tubercular lesion on radiography of the chest.

c. Evidence of confirmed active extrapulmonary tuberculosis (either by microscopic examination or by culture of the affected tissue for M. tuberculosis).

4. *Exclusion of Other Uveitis Entities*

In the geographic regions where tuberculosis is low in incidence, other causes of uveitis must be excluded by various laboratory investigations including serology for syphilis, toxoplasmosism and others.

5. *Therapeutic Test*

A positive response to 4-drug ATT (isoniazid, rifampicin, ethambutol, and pyrazinamide) over a period of 4 to 6 weeks. Therapeutic trial with single drug isoniazid should be avoided due to risk of development of resistance. It is important to refer such a patient to a TB expert who can initiate and monitor the treatment. The therapeutic response to ATT in the eye should, however be evaluated by the ophthalmologist.

_____________________________________________
